# Accuracy of performance-test linking based on a many-facet Rasch model

**DOI:** 10.3758/s13428-020-01498-x

**Published:** 2020-11-09

**Authors:** Masaki Uto

**Affiliations:** The University of Electro-Communications, Tokyo, Japan

**Keywords:** Performance assessment, Item response theory, Many-facet Rasch models, IRT linking, Test design, Rater effects, Educational measurement

## Abstract

Performance assessments, in which human raters assess examinee performance in practical tasks, have attracted much attention in various assessment contexts involving measurement of higher-order abilities. However, difficulty persists in that ability measurement accuracy strongly depends on rater and task characteristics such as rater severity and task difficulty. To resolve this problem, various item response theory (IRT) models incorporating rater and task parameters, including many-facet Rasch models (MFRMs), have been proposed. When applying such IRT models to datasets comprising results of multiple performance tests administered to different examinees, test linking is needed to unify the scale for model parameters estimated from individual test results. In test linking, test administrators generally need to design multiple tests such that raters and tasks partially overlap. The accuracy of linking under this design is highly reliant on the numbers of common raters and tasks. However, the numbers of common raters and tasks required to ensure high accuracy in test linking remain unclear, making it difficult to determine appropriate test designs. We therefore empirically evaluate the accuracy of IRT-based performance-test linking under common rater and task designs. Concretely, we conduct evaluations through simulation experiments that examine linking accuracy based on a MFRM while changing numbers of common raters and tasks with various factors that possibly affect linking accuracy.

## Introduction

With the increasing need for measuring higher-order abilities such as logical thinking and problem-solving, performance assessments, in which human raters assess examinee performance on practical tasks, have attracted attention (Rosen & Tager, 2014; Liu, Frankel, & Roohr, 2014; Bernardin, Thomason, Buckley, & Kane, 2016; Abosalem, 2016; Schendel & Tolmie, 2017; Uto & Ueno, 2018). Performance assessment has been applied to various formats, including essay-writing tests for college entrance examinations, speaking tests for language exams, report writing or programming assignments in learning situations, and objective-structured clinical examinations.

However, one limitation of performance assessments is that their accuracy for ability measurement strongly depends on rater and task characteristics such as rater severity and task difficulty (Kassim, 2011; Myford & Wolfe, 2003; Eckes, 2005; 2015; Bernardin et al., 2016). To resolve this problem, various item response theory (IRT) models incorporating parameters for rater and task characteristics have been proposed (Myford & Wolfe, 2003; Eckes, 2015; Uto & Ueno, 2018). The many-facet Rasch models (MFRMs) (Linacre, 1989) are the most popular IRT models with rater and task parameters, and various MFRM extensions have also been recently proposed (Patz & Junker, 1999; Patz, Junker, Johnson, & Mariano, 2002; Uto & Ueno, 2020; Uto, 2019). By considering rater and task characteristics, such IRT models can measure examinee abilities with higher accuracy than is possible with simple scoring methods based on point totals or averages (Uto & Ueno, 2020).

Actual testing situations often call for comparing the results of different performance tests administered to different examinees (Engelhard, 1997; Muraki, Hombo, & Lee, 2000). To apply IRT models in such cases, *test linking* is needed to unify the scale at which model parameters are estimated from individual test results. Performance-test linking generally requires some extent of overlap for examinees, tasks, and raters between tests (Engelhard, 1997; Linacre, 2014; Eckes, 2015; Ilhan, 2016). Specifically, tests must be designed such that at least two of the three facets (examinees, tasks, and raters) are partially common (Engelhard, 1997; Linacre, 2014). Test linking with common raters and tasks is generally preferred in practice because test designs that assume common examinees induce a higher response burden, potentially influencing practices or learning effects (Engelhard, 1997; Izumi, Yamano, Yamada, Kanamori, & Tsushima, 2012; Linacre, 2014).

The accuracy of linking under designs with common raters and tasks is highly reliant on the numbers of common raters and tasks, with higher numbers generally improving linking accuracy (Linacre, 2014). However, increasing numbers of common raters increases their assessment workload, while increasing numbers of common tasks might reduce test reliability owing to the potential for exposure of task contents (Way, 1998; van der Linden & Pashley, 2000; van der Linden, 2005a; Ishii, Songmuang, & Ueno, 2014). It is thus necessary to design tests such that numbers of common raters and tasks are minimized while retaining high test-linking accuracy.

However, the numbers of common raters and tasks required for ensuring high accuracy of test linking remains unclear. Linacre (2014) suggested that at least five common raters and five common tasks are required to obtain sufficient test linking accuracy for MFRMs, but provided no basis for justifying this standard. Previous research related to traditional IRT-based linking for objective tests has reported that the required extent of commonality depends on the distributions of examinee ability and item characteristics, the numbers of examinees and items, and the accuracy of model parameter estimation (Kilmen and Demirtasli, 2012; Uysal & Ibrahim, 2016; Joo, Lee, & Stark, 2017). These findings suggest that the extent to which IRT-based performance-test linking requires common raters and tasks depends basically on the following factors:

1. distributions of examinee ability and characteristics of raters and tasks,
2. numbers of examinees, raters, and tasks, and
3. rates of missing data.

We assume the rate of missing data as a factor affecting linking accuracy because it affects parameter estimation accuracy (Uto, Duc Thien, & Ueno, 2020). Note that missing data occur in practice because few raters are generally assigned to individual evaluation targets to lessen raters’ scoring burdens.

Thus, this study empirically evaluates the effects of the above three factors on the accuracy of IRT-based performance-test linking under designs with common raters and tasks. Concretely, this study conducts simulation experiments that examine test-linking accuracy while varying the above three factors and numbers of common raters and tasks. Although there are various IRT models with rater and task parameters, as mentioned above, this study focuses on the most popular MFRM. From experimental results, we discuss the numbers of common raters and tasks required for accurate linking in various test settings.

## Performance assessment data

This study assumes rating data *U* obtained from a performance test result as a set of ratings *x*_*ijr*_, assigned by rater $r\in \mathcal {R} = \{1,\ldots ,R\}$ to the performance of examinee $j \in \mathcal {J} = \{1,\cdots ,J\}$ on performance task $i\in \mathcal {I} = \{1,\ldots ,I\}$, where $\mathcal {R}$, $\mathcal {J}$, and $\mathcal {I}$ indicate sets of raters, examinees, and tasks, respectively. Concretely, the data can be defined as

$$ {U} = \{ x_{ijr} \in \mathcal{K} \cup \{-1\} \mid i \in \mathcal{I}, j \in \mathcal{J}, r \in \mathcal{R}\},  $$

where $\mathcal {K} = \{1,\ldots ,K\}$ is the rating categories, and *x*_*ijr*_ = − 1 indicates missing data. Missing data occur in actual performance assessments because few raters are generally assigned to individual evaluation targets to lessen the scoring burden (Engelhard, 1997; Eckes, 2015; Ilhan, 2016; Uto et al., 2020). A typical rater assignment strategy is the *rater-pair design* (Eckes, 2015), which assigns two raters to each evaluation target. Table 1 shows an example rater-pair design. In the table, checkmarks indicate an assigned rater, and blank cells indicate that no rater was assigned. In this Table 1, raters 1 and 2 are assigned to the performance of examinee 1 on task 1, while raters 3 and 4 are assigned to the performance of examinee 2. Rater-pair design greatly reduces raters’ scoring burden relative to the case where all raters evaluate all performances, but generally decrease the accuracy of examinee ability measurements.

**Table 1:** Example of rater-pair design

	Task 1				Task 2				Task 3			
Rater	1	2	3	4	1	2	3	4	1	2	3	4
Examinee 1	✓	✓			✓			✓	✓		✓	
Examinee 2			✓	✓		✓	✓			✓		✓
Examinee 3	✓		✓		✓	✓			✓			✓
Examinee 4		✓		✓			✓	✓		✓	✓	

This study assumes application of IRT to these performance assessment data.

## Item response theory for performance assessment

IRT is a testing theory based on a mathematical model (Lord, 1980). With the spread of computer testing, it has been widely applied in various testing situations. In IRT, examinee responses to test items are expressed as a probabilistic model defined according to examinees’ abilities and item characteristics, such as difficulty and discrimination power. IRT can thus estimate examinee abilities while considering test item characteristics. IRT has been used as the basis for current test theories such as automatic uniform test assembly and adaptive testing (van der Linden, 2005b; Songmuang & Ueno, 2011; Ishii et al., 2014).

Well-known IRT models that are applicable to ordered-categorical data like performance assessment data include the rating scale model (Andrich, 1978), the partial credit model (Masters, 1982), the graded response model (Samejima, 1969) and the generalized partial-credit model (Muraki, 1997). Such traditional IRT models are applicable to two-way data consisting of *examinees* × *test items*. However, these cannot be directly applied to three-way data comprising *examinees* × *raters* × *tasks* from performance assessments 1. Many IRT models with rater and task parameters have been proposed to address this problem (Myford & Wolfe, 2003; Eckes, 2015; Uto & Ueno, 2018).

MFRMs (Linacre, 1989) are the most popular IRT models with rater and task parameters, and have long been used to analyze performance assessment data (Myford & Wolfe, 2003; Eckes, 2005; Eckes, 2015; Chan, Bax, & Weir, 2017; Tavakol & Pinner, 2019). There are several MFRM variants (Eckes, 2015), but the most representative modeling defines the probability that $x_{ijr} =k \in \mathcal {K}$ as

1 $$ P_{ijrk} = \frac{\exp {\sum}_{m=1}^{k}\left[ \theta_{j}-\beta_{i}-\gamma_{r} - d_{m} \right]}{{\sum}_{l=1}^{K} \exp {\sum}_{m=1}^{l}\left[\theta_{j}-\beta_{i}-\gamma_{r} - d_{m} \right]},  $$

where *𝜃*_*j*_ is the latent ability of examinee *j*, *β*_*i*_ is the difficulty of task *i*, *γ*_*r*_ is the severity of rater *r*, and *d*_*k*_ is a category parameter that denotes the difficulty of transition between scores *k* − 1 and *k*. For model identification, *γ*_1_ = 0, *d*_1_ = 0, and ${\sum }_{k=2}^{K} d_{k} = 0$ are assumed. See Refs. (Eckes, 2015; Uto & Ueno, 2018; 2020) for details of the rater and task parameter interpretation.

This study focuses on this MFRM because it is the most popular model, but note that various MFRM extensions have been recently proposed (Patz & Junker, 1999; Patz et al., 2002; Uto & Ueno, 2020; Uto, 2019).

## IRT-based performance-test linking

MFRM and its extended models allow measuring examinee ability while considering rater and task characteristics, providing higher accuracy than simple scoring methods such as total or average scores (Uto & Ueno, 2018; 2020). Also, the model provides rater and task parameter estimates, helping test administrators to objectively analyze rater and task characteristics (Eckes, 2005; Myford & Wolfe, 2000; Chan et al., 2017; Tavakol & Pinner, 2019). Therefore, practical application of these models to actual performance assessments is beneficial.

Actual testing scenarios often require comparison of results from multiple performance tests applied to different examinees (Muraki et al., 2000). Applying IRT models to such cases generally requires test linking, in which model parameters estimated from individual test results use the same scale. Although linking is not required when equal between-test distributions of examinee abilities and characteristics of raters and tasks can be assumed (Linacre, 2014), actual testing situations will not necessarily satisfy such assumptions, and thus require test linking.

Although various situations require linking, this study assumes situations where the parameters for a newly conducted performance test use already estimated parameter scales from a previous performance test. Below, we designate the newly conducted performance test as the *new test*, and the test for determining the scales of parameters as the *base test*.

**Fig. 1:**
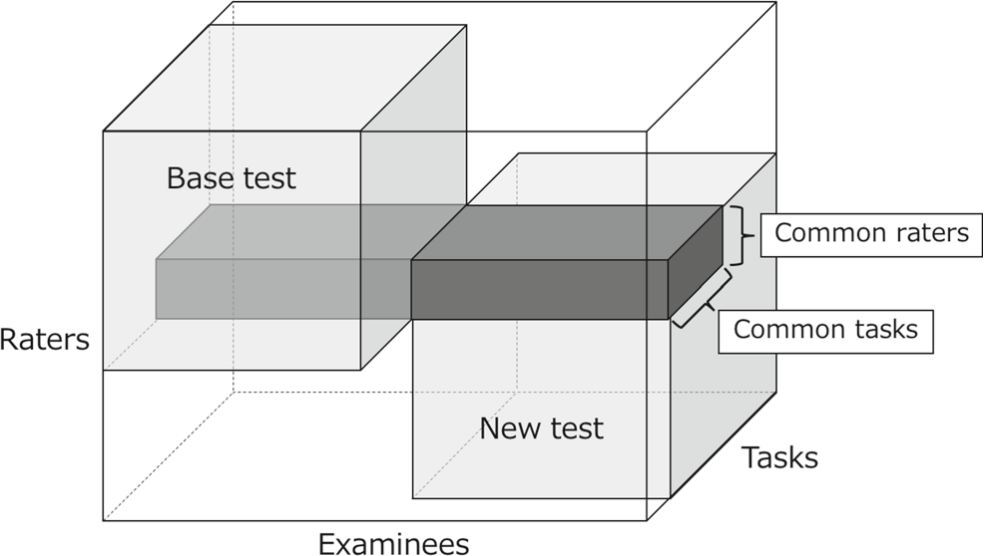
Linking design using common raters and common tasks

One representative method of test linking is to design tests such that some raters and tasks are shared between tests, as described in “Introduction” (Engelhard, 1997; Linacre, 2014; Eckes, 2015; Ilhan, 2016). Figure 1 shows the data structure for two performance tests with common raters and tasks. As defined in “Performance assessment data”, performance assessment data are three-way data consisting of *examinees* × *raters* × *tasks*, and so are represented in the figure as a three-dimensional array. In the figure, colored regions indicate available data, while other regions represent missing data. As the figure shows, data are collected such that raters and tasks are partially shared between two tests. In this design, parameters for the new test are expected to be on the same scale as those for the base test by estimating them while fixing parameters for common raters and tasks that are estimated in advance from the base test data (Linacre, 2014; Eckes, 2015; Ilhan, 2016). This linking design is a variant of the *nonequivalent groups with anchor test design* (Dorans, Pommerich, & Holland, 2007) or the *common item nonequivalent groups design* (Kolen & Brennan, 2014), typical designs used for objective test linking. In our design, common raters and common tasks take the role of an anchor test or common items. Furthermore, the linking method used here is a simple extension of the *fixed common item parameters method*, a common method in IRT-based objective test linking (Arai & Mayekawa, 2011; Jodoin, Keller, & Swaminathan, 2003; Li, Tam, & Tompkins, 2004)because it estimates the new test parameters while fixing parameters for common raters and tasks.

In this design, linking accuracy is strongly dependent on the numbers of shared raters and tasks (Linacre, 2014). Although increasing these numbers generally improves test-linking accuracy, these numbers should be kept as low as possible while maintaining required test linking accuracy, as described in “Introduction”. However, the required numbers of common raters and tasks for ensuring high-accuracy test linking remain unknown. As discussed in “Introduction”, the extent to which common raters and tasks are required for performance-test linking would typically depend on the three factors, namely,

1. distributions of examinee ability and characteristics of raters and tasks,
2. numbers of examinees, raters, and tasks, and
3. rates of missing data. Therefore, in this study we examined the numbers of common raters and tasks necessary for high-accuracy test linking while changing settings for these three factors.

Ideally, evaluation experiments should be conducted using actual data. However, designing and executing actual tests for various settings would entail huge costs and time. In this study, therefore, we evaluated test-linking accuracy by simulation experiments, as in previous studies of IRT-based objective test linking (Fujimori, 1998; Arai & Mayekawa, 2011; Kilmen & Demirtasli, 2012; Uysal & Ibrahim, 2016).

## Linking accuracy criteria

This study evaluates MFRM-based performance-test linking accuracy through the following simulation procedure, which is based on a typical experimental method for evaluating IRT-based objective test linking accuracy (Lee & Ban, 2009; Arai & Mayekawa, 2011; Kilmen & Demirtasli, 2012; Uysal & Ibrahim, 2016).

1. Assuming a *base test* with *I* tasks, *J* examinees, and *R* raters, generate true values for MFRM parameters for the base test with distributions
  2 $$ \beta_{i}, \gamma_{r}, d_{k}, \theta_{j} \sim N(0.0,1.0) , $$
  where *N*(*μ*,*σ*^2^) represents the normal distribution with mean *μ* and standard deviation *σ*. Note that *d*_*k*_ values must satisfy the constraints *d*_1_ = 0, and ${\sum }_{k=2}^{K} d_{k} = 0$, as explained in “Item response theory for performance assessment”. In addition, the values for {*d*_*k*_∣*k* ≥ 2} are expected to be monotonically ascending in practice. Therefore, we sorted the generated values for {*d*_*k*_∣*k* ≥ 2} in ascending order, then linearly transformed these values such that their total value becomes zero. We also set *d*_1_ = 0. In this study, we set the number of rating categories as *K* = 5.
2. Similarly, assuming a *new test* with *I*, *J*, and *R*, generate true values for MFRM parameters for the new test from arbitrary distributions, which differ from the above distributions.
3. Establish *C*_*R*_ common raters and *C*_*I*_ common tasks between the tests. Specifically, parameter values for *C*_*R*_ raters and *C*_*I*_ tasks selected from the new test are replaced with parameter values for *C*_*R*_ raters and *C*_*I*_ tasks, which are randomly selected from the base test. From this procedure, *C*_*R*_ raters and *C*_*I*_ tasks from the base test are incorporated into the new test as common raters and tasks.
4. Sample rating data for the new test following MFRM given the model parameters generated through the above procedures.
5. Estimate parameters for the new test from the generated data by fixing the parameters for common raters and tasks, then calculate the root mean square error (RMSE) between the estimates and the true parameter values. We use the expected a posteriori estimation by Markov-chain Monte Carlo (Uto & Ueno, 2020) for the parameter estimation, given the distributions of Eq. 2 as the prior distributions. In the parameter estimation, the constraint *γ*_1_ = 0, which is assumed for model identification, is omitted because fixing the parameters for common raters and tasks can resolve the model identification problem.
6. After repeating the above procedures 30 times, calculate average RMSE values for each commonality number.

In this experiment, insufficient numbers for common raters and tasks will increase parameter estimation error for the new test because the new test’s parameters are estimated based on the prior distributions of Eq. 2, which differ from the distributions generating their true parameter values. Conversely, sufficient numbers decrease parameter estimation error because the fixed parameters for common raters and tasks, which are generated following the distributions of Eq. 2, serve as the basis for adjusting the new test’s parameters to their true locations. High-accuracy test linking is thus realized under given numbers of common raters *C*_*R*_ and tasks *C*_*I*_ if the averaged RMSE value obtained from the above experiment is sufficiently small.

To judge from the RMSE value whether a new test is linked with sufficient accuracy, we need to establish a threshold RMSE value. To do so, we conducted a similar experiment to the above, in which the parameter distributions of Eq. 2 are used as the distributions for the new test in experimental procedure 2. In this case, because the parameter distributions are equal for the base test and the new test, the new test is completely linked regardless of the presence or absence of common raters and tasks, as described in “IRT-based performance-test linking”. We can thus regard the RMSE value obtained from this experiment as a threshold value for determining whether test linking has high accuracy. Specifically, we define the threshold *δ* = *μ*_*e*_ + 2*σ*_*e*_, where *μ*_*e*_ and *σ*_*e*_ are the average and standard deviation of RMSEs obtained from the 30 repetitions in procedure 6. Note that we allow up to 2*σ*_*e*_ deviation from the average value *μ*_*e*_ because the RMSE can vary for each repetition of the experiment, depending on the generated data or true parameters, and because 95*%* of such varying RMSE values fall within that range.

This study thus assumes that high-accuracy test linking is realized if the average RMSE value obtained under a target setting is lower than the corresponding threshold value *δ*.

Note that alternative approaches for evaluating linking accuracy, such as that in Linacre (1998), may be possible if we use other linking methods, such as scale transformation methods with separate calibration or concurrent calibration methods (Kolen and Brennan, 2014; Arai & Mayekawa, 2011; Jodoin et al., 2003; Ryan & Rockmann, 2009), instead of the fixed rater and task parameters method.

## Experiments

In this section, we present experimental results from changing the settings for the three factors described above. In the experiments, we mainly examine small- or mid-scale test settings in which the maximum number of examinees is 100 because it is difficult to examine various conditions for large-scale settings due to the high computational complexity of our experiment. “Large-scale examples” shows some results for large-scale settings. Furthermore, in “Effect of changes in characteristics of common raters and tasks” and “Use of other error indices to calculate linking accuracy criteria”, we discuss two issues related to our experimental assumptions and procedures. Java programs developed for the following experiments are published in a GitHub repository. See *Open Practices Statement* for details.

### Evaluating effects of between-test distribution differences

This subsection describes the effects on test linking accuracy of varying distributions of examinee ability and characteristics of raters and tasks for a new test. Specifically, we conducted the experiment described in “Linking accuracy criteria” while varying parameter distributions of the new test following the four conditions in Table 2. Here, *distribution 1* represents the case in which only the ability distribution differs from that of the base test, and *distribution 2* describes the case of reduced difference in the ability distribution. *Distribution 3* and *distribution 4* are cases in which both the examinee ability distribution and the rater or task characteristic distribution differ.

**Table 2:** Parameter distributions for the new test

	*𝜃*_*j*_	*β*_*i*_	*γ*_*r*_	*d*_*k*_
Distribution 1	*N*(− 0.5, 1.0)	*N*(0.0, 1.0)	*N*(0.0, 1.0)	*N*(0.0, 1.0)
Distribution 2	*N*(− 0.2, 1.0)	*N*(0.0, 1.0)	*N*(0.0, 1.0)	*N*(0.0, 1.0)
Distribution 3	*N*(− 0.5, 1.0)	*N*(0.5, 1.0)	*N*(0.0, 1.0)	*N*(0.0, 1.0)
Distribution 4	*N*(− 0.5, 1.0)	*N*(0.0, 1.0)	*N*(0.5, 1.0)	*N*(0.0, 1.0)

The case of distribution 1, where the mean value of the ability distribution between tests varies by 0.5, can be regarded as a realistic situation in which linking is difficult. This is because when we randomly sample *N* data from a larger population following a standard normal distribution, the standard deviation of the sampling distribution’s mean (the *standard error of the mean*, SEM) is estimable as $1/\sqrt {N}$. Thus, for example, when 100 examinees take a test, the SEM can be estimated as 0.1. In this case, the 98.8% confidence interval of the mean values is about the *meanvalue* ± 0.25 (corresponding to the ± 2.5 SEM range), meaning that situations where the between-test distribution mean difference exceeds 0.5 rarely happen.

This study thus regards distribution 1 as a baseline setting because the results from this setting are expected to provide a basis for the maximum numbers of required common raters and tasks. Note that test linking becomes more difficult for distributions 3 or 4 because both the examinee ability distribution and the rater or task characteristic distribution differ. We do not regard this as a baseline setting, however, because in practice test administrators manage multiple tests such that rater and task characteristics are as similar as possible to assure fairness, making differences in rater and task characteristic distributions between tests relatively small.

In this experiment, we fixed factors other than the new test distributions. Specifically, we set *J* = 100, *I* = 10, and *R* = 10. This experiment was conducted assuming no missing data, meaning all raters grade all examinees’ performance on all tasks.

Table 3 shows the results. Values in parentheses indicate the threshold *δ*. Bold text indicates that the RMSE value is lower than the corresponding threshold value *δ*, meaning that high-accuracy linking is achieved. Note that in Table 3, the threshold value *δ* is the same for all distributions because *δ* depends only on the data size, which is the same for all distribution settings in this experiment.

**Table 3:** Experimental results for different parameter distributions

*C*_*R*_	*C*_*I*_= 1	*C*_*I*_= 2	*C*_*I*_= 3	*C*_*I*_= 4	*C*_*I*_= 5
Distribution 1					
1	.1538(.1476)	**.1377**(.1435)	**.1421**(.1433)	**.1380**(.1426)	**.1383**(.1421)
2	.1483(.1423)	**.1273**(.1340)	**.1275**(.1399)	**.1327**(.1427)	**.1261**(.1356)
3	.1574(.1461)	**.1353**(.1373)	**.1268**(.1358)	**.1274**(.1336)	**.1220**(.1371)
4	.1420(.1360)	**.1250**(.1404)	**.1203**(.1421)	**.1265**(.1343)	**.1189**(.1336)
5	.1469(.1458)	**.1270**(.1346)	**.1210**(.1455)	**.1254**(.1350)	**.1244**(.1450)
Distribution 2					
1	**.1275**(.1476)	**.1183**(.1435)	**.1175**(.1433)	**.1194**(.1426)	**.1206**(.1421)
2	**.1300**(.1423)	**.1201**(.1340)	**.1242**(.1399)	**.1184**(.1427)	**.1176**(.1356)
3	**.1224**(.1461)	**.1195**(.1373)	**.1178**(.1358)	**.1238**(.1336)	**.1174**(.1371)
4	**.1195**(.1360)	**.1224**(.1404)	**.1160**(.1421)	**.1181**(.1343)	**.1168**(.1336)
5	**.1277**(.1458)	**.1180**(.1346)	**.1203**(.1455)	**.1188**(.1350)	**.1148**(.1450)
Distribution 3					
1	.1679(.1476)	.1464(.1435)	**.1432**(.1433)	**.1424**(.1426)	**.1406**(.1421)
2	.1596(.1423)	.1406(.1340)	**.1346**(.1399)	**.1279**(.1427)	**.1327**(.1356)
3	.1544(.1461)	**.1354**(.1373)	**.1343**(.1358)	**.1300**(.1336)	**.1254**(.1371)
4	.1462(.1360)	**.1340**(.1404)	**.1307**(.1421)	**.1263**(.1343)	**.1280**(.1336)
5	**.1432**(.1458)	**.1297**(.1346)	**.1309**(.1455)	**.1282**(.1350)	**.1243**(.1450)
Distribution 4					
1	.1605(.1476)	.1513(.1435)	.1491(.1433)	**.1396**(.1426)	**.1359**(.1421)
2	.1473(.1423)	.1435(.1340)	**.1350**(.1399)	**.1348**(.1427)	**.1274**(.1356)
3	.1531(.1461)	**.1357**(.1373)	**.1280**(.1358)	**.1266**(.1336)	**.1272**(.1371)
4	.1470(.1360)	**.1287**(.1404)	**.1304**(.1421)	**.1267**(.1343)	**.1242**(.1336)
5	.1501(.1458)	**.1320**(.1346)	**.1238**(.1455)	**.1232**(.1350)	**.1256**(.1450)

The table shows that high-accuracy linking tends to be realized when numbers of common raters or tasks increase, as expected.

According to the results for distribution 1, high-accuracy linking is achieved in all cases where *C*_*I*_ ≥ 2. Further, the results for distribution 2 show that numbers of required common raters and tasks decrease with reduced difference in between-test ability distributions. Specifically, in the distribution 2 case, adequate test linking is possible with one common rater and one common task. The results of distributions 3 and 4 show that numbers of required commonality increase when the distributions for rater and task parameters differ among tests. These results suggest that we need *C*_*I*_ + *C*_*R*_ = 5 or 6 for the distribution 3 and 4 cases.

As mentioned in “Introduction”, Linacre (2014) suggested that at least five common raters and five common tasks (namely, *N*_*R*_ ≥ 5 and *N*_*I*_ ≥ 5) are required to obtain sufficient test linking accuracy. However, our experimental results show that these numbers can be substantially reduced not only for realistic cases where ability distributions differ among tests, but also for the relatively rare cases where rater and task characteristics distributions differ too.

### Evaluating effects of numbers of examinees, tasks, and raters

This section presents an analysis of the effects of numbers of examinees, tasks, and raters on test linking accuracy. Specifically, we examined the following four settings:

- *J* = 50, *I* = 5, *R* = 5
- *J* = 100, *I* = 5, *R* = 5
- *J* = 100, *I* = 10, *R* = 5
- *J* = 100, *I* = 5, *R* = 10

In this experiment, we fixed the parameter distribution for the new test to distribution 1 in Table 2. As in the previous experiment, this experiment assumes there are no missing data.

Table 4 shows the results. Note that *δ* values in parentheses vary for each setting, unlike those in Table 3, because *δ* depends on the data size, which differs for each setting.

**Table 4:** Experimental results for different numbers of examinees, tasks, and raters

*C*_*R*_	*C*_*I*_= 1	*C*_*I*_= 2	*C*_*I*_= 3	*C*_*I*_= 4	*C*_*I*_= 5
J = 50, I = 5, R = 5					
1	**.2829**(.2881)	**.2754**(.3038)	**.2681**(.2826)	**.2782**(.3005)	**.2519**(.2811)
2	**.2716**(.3044)	**.2786**(.2794)	**.2513**(.2808)	**.2543**(.2711)	**.2541**(.2820)
3	**.2739**(.2822)	**.2399**(.2920)	**.2263**(.3002)	**.2484**(.2782)	**.2371**(.2798)
4	**.2689**(.2859)	**.2482**(.2785)	**.2433**(.2642)	**.2406**(.2746)	**.2366**(.2732)
5	**.2746**(.3104)	**.2658**(.2741)	**.2466**(.2873)	**.2304**(.2823)	**.2372**(.2858)
J = 100, I = 5, R = 5					
1	.3025(.2942)	**.2691**(.2814)	**.2554**(.2921)	**.2640**(.2878)	**.2673**(.2829)
2	.2693(.2685)	**.2584**(.2671)	**.2479**(.2764)	**.2544**(.2623)	**.2501**(.2720)
3	**.2740**(.2852)	**.2581**(.2837)	**.2461**(.2684)	**.2566**(.2815)	**.2431**(.2705)
4	**.2778**(.2783)	**.2496**(.2840)	**.2366**(.2806)	**.2533**(.2861)	**.2401**(.2909)
5	**.2626**(.2722)	**.2545**(.2698)	**.2475**(.2840)	**.2514**(.2865)	**.2506**(.2739)
J = 100, I = 10, R = 5					
1	.2187(.2066)	.1995(.1966)	.2039(.1908)	.1995(.1911)	.1985(.1938)
2	.2048(.2026)	**.1890**(.1981)	**.1887**(.2021)	**.1803**(.1921)	**.1870**(.1918)
3	.2065(.1986)	**.1952**(.1985)	**.1790**(.1944)	**.1798**(.1975)	**.1774**(.2153)
4	**.1937**(.2035)	**.1872**(.2094)	**.1716**(.1968)	**.1750**(.1951)	**.1746**(.1970)
5	**.1934**(.1984)	**.1803**(.1956)	**.1742**(.2101)	**.1740**(.2023)	**.1746**(.1910)
J = 100, I = 5, R = 10					
1	.2212(.2099)	**.1915**(.2113)	**.1908**(.2011)	**.1864**(.1977)	**.1921**(.1953)
2	.2198(.2007)	**.1879**(.2017)	**.1848**(.1903)	**.1783**(.1867)	**.1799**(.1912)
3	.2142(.2040)	**.1808**(.2078)	**.1785**(.1978)	**.1735**(.1932)	**.1773**(.1916)
4	.1955(.1945)	**.1786**(.1946)	**.1787**(.1978)	**.1743**(.2018)	**.1684**(.1927)
5	**.2059**(.2068)	**.1794**(.1971)	**.1763**(.1917)	**.1815**(.1913)	**.1735**(.1998)

Table 4 and the results for distribution 1 in Table 3 show that the extent of required commonality for accurate linking increases with increased numbers of examinees, raters, and tasks. According to these results, adequate linking is possible with only one common rater and one common task for small-scale settings, while about two common raters and two common tasks are required when the numbers of examinees, raters, and tasks increase.

Although the impact of changes in numbers of examinees, raters, and tasks on linking accuracy is not so large for these small- or mid-scale settings, these results suggest that the extent of required commonality may further increase for large-scale scenarios. We consider such cases in “Large-scale examples”.

### Evaluating effects of missing data

The above experiments assumed that all raters grade all examinees’ performance on all tasks. In actual scenarios, however, only a few raters are assigned for each performance to lower the scoring burden, as described in “Performance assessment data”. In such cases, large amounts of missing data occur, generally lowering parameter estimation accuracy. This decrease in parameter estimation accuracy is known to lower test linking accuracy (Izumi et al., 2012). This section, therefore, evaluates how missing data affect test linking accuracy.

In this study, we assume that rater assignments follow a judge-pair design, described in “Performance assessment data” as a typical rater assignment strategy. Ilhan (2016) proposed an algorithm for generating rater-pair designs under conditions where test linking is possible. Specifically, this algorithm first lists all rater pairs, then sequentially allocates evaluation targets to each rater pair. We generalized this algorithm so that three or more raters can be assigned. Algorithm 1 shows pseudocode for the generalized algorithm, with *N*_*R*_ indicating the number of raters assigned to each evaluation target, where *R* ≥ *N*_*R*_ ≥ 2. We call this rater assignment design *rater set design*. 
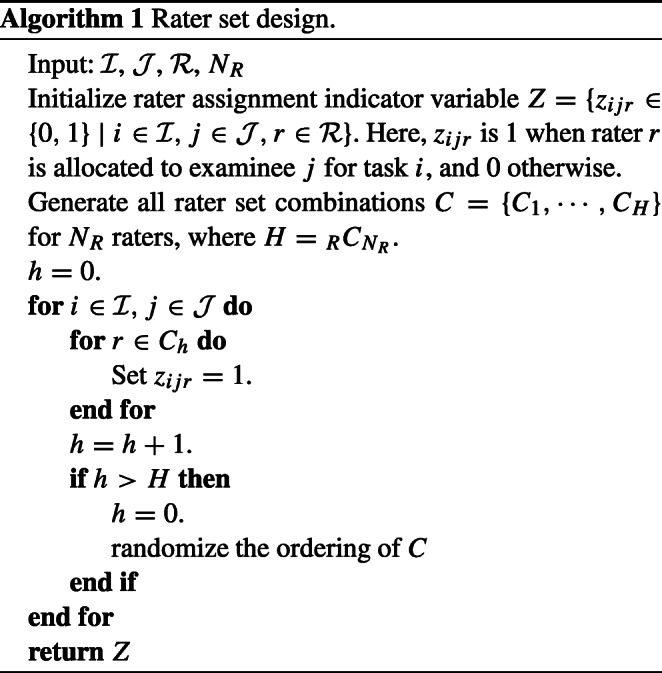
 We conducted the experiment described in “Linking accuracy criteria” while applying the rater set design. Concretely, after generating the rating data in experimental procedure 4 of “Linking accuracy criteria”, we omit ratings for each performance to which no raters are assigned in the rater set design created by Algorithm 1. We conducted this experiment under the following settings while fixing *J* = 100 and*I* = 10.

- *R* = 5, *N*_*R*_ = 2 (60*%* missing)
- *R* = 10, *N*_*R*_ = 3 (70*%* missing)
- *R* = 10, *N*_*R*_ = 2 (80*%* missing)

Here, the rate of missing data is calculable as $\left [1-(N_{R}/R)\right ]\times 100$. In this experiment, we used distribution 1 in Table 2 for the new test.

Table 5 shows the results, which confirm that the extent of commonality required for accurate linking tends to increase with higher rates of missing data. Specifically, the results suggest that adequate test linking is impossible with *C*_*I*_ = 2 and/or *C*_*R*_ = 2, unlike the case of no missing data, and that we need about *C*_*I*_ + *C*_*R*_ = 6 at minimum for situations with 80% missing data. Even so, note that these numbers are still smaller than those suggested by Linacre (2014).

**Table 5:** Experimental results for different rates of missing data

*C*_*R*_	*C*_*I*_= 1	*C*_*I*_= 2	*C*_*I*_= 3	*C*_*I*_= 4	*C*_*I*_= 5
R = 5, *N*_*R*_= 2 (60% missing)					
1	.3616(.3082)	.3180(.2990)	**.3099**(.3155)	**.2900**(.3097)	**.2990**(.3018)
2	.3458(.3048)	.3123(.2981)	**.2933**(.2958)	**.2892**(.3069)	**.2808**(.3090)
3	.3291(.3088)	.3064(.2911)	**.2917**(.2923)	**.2789**(.3039)	**.2721**(.3106)
4	.3317(.3109)	.3032(.2856)	**.2856**(.3064)	**.2715**(.3063)	**.2680**(.2875)
5	.3189(.2966)	.2998(.2927)	**.2945**(.2967)	**.2885**(.2914)	**.2642**(.3037)
R = 10, *N*_*R*_= 3 (70% missing)					
1	.3187(.2510)	.2943(.2592)	.2795(.2431)	.2722(.2386)	.2733(.2511)
2	.2792(.2519)	.2610(.2368)	.2545(.2503)	**.2400**(.2477)	**.2443**(.2502)
3	.2777(.2584)	.2434(.2319)	**.2347**(.2478)	**.2330**(.2589)	**.2365**(.2464)
4	.2869(.2507)	.2471(.2463)	**.2318**(.2554)	**.2259**(.2529)	**.2215**(.2426)
5	.2803(.2462)	.2537(.2345)	**.2318**(.2495)	**.2280**(.2349)	**.2267**(.2501)
R = 10, *N*_*R*_= 2 (80% missing)					
1	.3795(.3128)	.3278(.2941)	.3399(.2897)	.3260(.2842)	.3187(.2998)
2	.3459(.3084)	.3127(.3036)	.3081(.2863)	**.3004**(.3010)	**.2915**(.2950)
3	.3541(.2898)	.3091(.2901)	.2992(.2968)	**.2884**(.2905)	**.2821**(.2899)
4	.3420(.3033)	.3141(.2985)	**.2833**(.2857)	**.2756**(.3059)	**.2798**(.2939)
5	.3488(.3002)	.3074(.2968)	**.2780**(.2976)	**.2796**(.2965)	**.2821**(.3066)

The factor inducing decreased test-linking accuracy would be a substantial decrease in parameter estimation accuracy due to high rates of missing data. Indeed, our experimental results indicate that the RMSE tends to increase as the rate of missing data increases. For example, Table 3 shows that the RMSE with *J* = 100, *I* = 10, *R* = 10, *C*_*I*_ = 1, and *C*_*R*_ = 1 is 0.1543 with no missing data, while Table 5 shows that the RMSE under the same settings is 0.3795 with 80% missing data.

These results also suggest that the required extent of commonality may further increase under large-scale test settings because the rate of missing data can increase. The increase in missing data is because the total number of raters generally increases with the increase in examinees, but the number of assigned raters for each evaluation target is difficult to increase. The next subsection presents the results for large-scale settings with a higher rate of missing data.

### Large-scale examples

The above experiments involved small- or mid-scale test settings in which the maximum number of examinees is 100 because examining various factors in large-scale settings incurs extremely high computational costs. However, as mentioned in “Evaluating effects of numbers of examinees, tasks, and raters” and “Evaluating effects of missing data”, increased scales might affect the required numbers of common raters and tasks. This section therefore presents examples of test linking results for large-scale test settings with the rater set design. Concretely, we conducted the same experiment as above with *J* = 1000, *I* = 5, and *R* = 20, applying the rater set design with *N*_*R*_ = 2 or 4. Note that we increased the number of raters because this would be performed in practice to lower the scoring burden for the increased number of examinees, as mentioned in “Evaluating effects of missing data”. Moreover, we set *I* = 5 to reduce computational costs, although the number of tasks in a test may also increase in large-scale settings.

Table 6 shows the results. Unlike in the case of the previous experiments, these experiments were conducted for *C*_*R*_ ∈{1,2,3,4,5,6,7,8,9,10}, due to the increased number of raters.

**Table 6:** Experimental results for large-scale settings

*C*_*R*_	*C*_*I*_= 1	*C*_*I*_= 2	*C*_*I*_= 3	*C*_*I*_= 4	*C*_*I*_= 5
J = 1000, I = 5, R = 20, *N*_*R*_= 2 (90% missing)					
1	.5310(.3841)	.5220(.3920)	.5263(.4061)	.5242(.3872)	.5177(.4076)
2	.5078(.3883)	.4906(.4007)	.4764(.3847)	.4814(.3873)	.4800(.4074)
3	.4929(.3993)	.4641(.3930)	.4480(.3919)	.4587(.3997)	.4525(.3928)
4	.4835(.4023)	.4525(.3910)	.4314(.3858)	.4340(.3956)	.4352(.4141)
5	.4751(.4070)	.4335(.4020)	.4432(.3905)	.4168(.4065)	.4201(.3980)
6	.4505(.3965)	.4347(.3962)	.4172(.3956)	.4195(.3996)	.4088(.3979)
7	.4526(.4071)	.4279(.4053)	.4109(.3962)	.4172(.3854)	**.3977**(.4042)
8	.4612(.3960)	.4130(.3974)	.4133(.3972)	.4024(.3960)	**.3932**(.4012)
9	.4599(.4153)	.4274(.3996)	**.3966**(.4020)	**.3931**(.3974)	**.3935**(.3975)
10	.4402(.3935)	.4250(.3894)	**.3953**(.3988)	**.3929**(.4055)	**.3859**(.3962)
J = 1000, I = 5, R = 20, *N*_*R*_= 4 (80% missing)					
1	.4184(.2883)	.3958(.2885)	.4042(.2871)	.3959(.2862)	.3917(.2823)
2	.3804(.2921)	.3563(.2956)	.3535(.2848)	.3539(.2960)	.3412(.2979)
3	.3509(.2952)	.3317(.3033)	.3312(.2830)	.3197(.2971)	.3264(.2824)
4	.3457(.2922)	.3159(.2889)	.3118(.2983)	.3029(.2881)	.3030(.2929)
5	.3454(.2904)	.3181(.3102)	.3004(.2856)	.2987(.2959)	.3015(.2918)
6	.3296(.2929)	.3064(.2937)	.2970(.2905)	.2943(.2914)	.2968(.2928)
7	.3236(.2929)	.2977(.2951)	**.2974**(.2987)	**.2905**(.2924)	**.2916**(.3050)
8	.3224(.2930)	.2966(.2928)	**.2856**(.2963)	**.2882**(.2971)	**.2827**(.2905)
9	.3206(.2886)	**.2934**(.2964)	**.2849**(.2891)	**.2893**(.2925)	**.2803**(.2932)
10	.3179(.3003)	**.2927**(.2981)	**.2837**(.2959)	**.2841**(.2921)	**.2822**(.2880)

Comparing these results with the previous results indicates a large increase in the required numbers of common raters and tasks. For example, when the rate of missing data is 80*%*, Table 5 shows that we need about *C*_*I*_ + *C*_*R*_ = 6 at minimum for *J* = 100, but Table 6 shows that *C*_*I*_ + *C*_*R*_ = 10 are required at minimum for the large-scale setting. This indicates that large increases of examinees and raters strongly affect the requirements for common raters and tasks. In addition, an increase in the number of tasks will also induce an increase in the required commonality, as demonstrated in “Evaluating effects of numbers of examinees, tasks, and raters”.

Table 6 also shows that the required numbers further increase as the rate of missing data increases, like in the experiment in “Evaluating effects of missing data”. Concretely, the results for a 90*%* rate of missing data show that the minimum required number is *C*_*I*_ + *C*_*R*_ = 12, which is larger than that suggested by Linacre (2014). In actual large-scale tests, the rate of missing data can be further increased with increased numbers of examinees and raters, so far more common raters and tasks might be required.

### Effect of changes in characteristics of common raters and tasks

The above experiments assumed that characteristics of common raters and tasks do not change across the base test and the new test. However, rater characteristics are known to often change across test administrations in practice (O’Neill and Lunz, 1997; Wolfe, Moulder, & Myford, 2001; Wesolowski, Wind, & Engelhard, 2017; Wind & Guo, 2019; Harik et al., 2009; Park, 2011), which is called *rater drift* (Harik et al., 2009; Park, 2011) or *differential rater functioning over time* (Wolfe et al., 2001). Similarly, in objective testing situations, item characteristics can also change due to educational practice or item exposure (Harik et al., 2009; Monseur & Berezner, 2007; Ryan & Rockmann, 2009), which is referred to as *item drift* or *item parameter drift*. This subsection therefore examines how changes in characteristics of common raters and tasks affect the linking accuracy.

To evaluate this, we calculated the linking accuracy while incorporating a deliberate fluctuation into the parameters of common raters and tasks before sampling rating data for the new test. Concretely, when we sample rating data for the new test in the procedure 4 described in “Linking accuracy criteria”, random values were added to the parameters of some common raters and tasks as fluctuations. Here, the numbers of common tasks and raters with the fluctuations were set to ⌊*C*_*I*_/2⌋ + *C*_*I*_*%*2 and ⌊(*C*_*R*_ − 1)/2⌋ + (*C*_*R*_ − 1)*%*2, respectively, where ⌊ ⌋ denotes floor function and *%* indicates the modulo operation. This means that we simulated situations where characteristics of about half of the common raters and tasks changed. The random fluctuation values were generated from a normal distribution with zero mean. The standard deviation for the fluctuation distributions was 0.05 for the common tasks and 0.10 for the common raters. These standard deviations were selected based on findings of empirical studies that examined item drifts (Monseur & Berezner, 2007) and rater drifts (O’Neill & Lunz, 1997; Wesolowski et al., 2017). Note that the parameters with such fluctuations were used only for sampling rating data. The original values of common raters and tasks were used as the fixed parameters for estimating the new test’s parameters. Also, the calculation procedures of the threshold values *δ* were completely the same as those described in “Linking accuracy criteria”.

Using this linking accuracy calculation method, we conducted the same experiment as that in “Evaluating effects of between-test distribution differences”. Table 7 shows the results. Comparing the results with Table 3, we can see that the required numbers of common raters and tasks tend to increase when the characteristics of common raters and tasks changed, although the increases are not dramatic. Concretely, according to the results, we need about one or two additional common raters and tasks to achieve accurate linking.

**Table 7:** Experimental results for different parameter distributions when characteristics of some common raters and tasks are changed

*C*_*R*_	*C*_*I*_= 1	*C*_*I*_= 2	*C*_*I*_= 3	*C*_*I*_= 4	*C*_*I*_= 5
Distribution 1					
1	.1629(.1476)	.1511(.1435)	.1460(.1433)	**.1357**(.1426)	**.1363**(.1421)
2	.1543(.1423)	.1491(.1340)	.1523(.1399)	**.1287**(.1427)	**.1284**(.1356)
3	.1468(.1461)	**.1349**(.1373)	**.1290**(.1358)	**.1296**(.1336)	**.1283**(.1371)
4	.1495(.1360)	**.1354**(.1404)	**.1238**(.1421)	**.1258**(.1343)	**.1266**(.1336)
5	.1508(.1458)	**.1317**(.1346)	**.1293**(.1455)	**.1284**(.1350)	**.1262**(.1450)
Distribution 2					
1	**.1299**(.1476)	**.1269**(.1435)	**.1228**(.1433)	**.1241**(.1426)	**.1234**(.1421)
2	**.1349**(.1423)	**.1231**(.1340)	**.1286**(.1399)	**.1281**(.1427)	**.1254**(.1356)
3	**.1347**(.1461)	**.1181**(.1373)	**.1210**(.1358)	**.1252**(.1336)	**.1247**(.1371)
4	**.1310**(.1360)	**.1285**(.1404)	**.1248**(.1421)	**.1229**(.1343)	**.1222**(.1336)
5	**.1240**(.1458)	**.1266**(.1346)	**.1214**(.1455)	**.1249**(.1350)	**.1195**(.1450)
Distribution 3					
1	.1620(.1476)	.1510(.1435)	.1464(.1433)	.1438(.1426)	.1445(.1421)
2	.1593(.1423)	.1434(.1340)	.1457(.1399)	**.1314**(.1427)	**.1306**(.1356)
3	.1489(.1461)	**.1338**(.1373)	**.1286**(.1358)	**.1320**(.1336)	**.1291**(.1371)
4	.1590(.1360)	**.1374**(.1404)	**.1292**(.1421)	**.1264**(.1343)	**.1325**(.1336)
5	**.1449**(.1458)	**.1334**(.1346)	**.1283**(.1455)	**.1324**(.1350)	**.1283**(.1450)
Distribution 4					
1	.1759(.1476)	.1519(.1435)	.1446(.1433)	.1507(.1426)	**.1374**(.1421)
2	.1521(.1423)	.1483(.1340)	.1459(.1399)	**.1354**(.1427)	**.1304**(.1356)
3	.1660(.1461)	.1436(.1373)	.1393(.1358)	**.1335**(.1336)	**.1321**(.1371)
4	.1597(.1360)	.1423(.1404)	**.1293**(.1421)	**.1271**(.1343)	**.1288**(.1336)
5	.1464(.1458)	**.1337**(.1346)	**.1348**(.1455)	**.1261**(.1350)	**.1287**(.1450)

These results suggest that in practice we may need to prepare slightly more common raters and tasks than as suggested in the earlier experiments as a safety margin to account for cases where rater and task characteristics change. Furthermore, the required numbers of common raters and tasks will likely further increase if changes in the characteristics of common raters and tasks are large, or if the numbers of raters and tasks whose characteristics changed increase. Conversely, these results mean that if we can carefully manage tests such that changes in rater and task characteristics become as small as possible, accurate linking can be realized with a smaller number of common raters and tasks.

### Use of other error indices to calculate linking accuracy criteria

As described in “Linking accuracy criteria”, this study defined linking accuracy criteria based on the RMSE between the parameter estimates and their true values. However, we may use alternative error indices, such as the average bias and the mean absolute error (MAE). Moreover, although this study calculated RMSE values over all parameters, these errors are calculable for only examinee ability estimates or rater/task parameter estimates. To examine how the error indices affect the results, we conducted the same experiment as that in “Evaluating effects of between-test distribution differences” using the absolute value of the average bias for examinee ability estimates.

Table 8 shows the results. Comparing the results with Table 3, the required numbers of common raters and tasks are almost the same. We also confirmed that several other indices, namely RMSE for examinee ability estimates, absolute average bias for all parameters, MAE for all parameters, and MAE for examinee ability estimates, suggest almost the same required numbers. Thus, we conclude that selection of error indices would not strongly affect the results.

**Table 8:** Experimental results for different parameter distributions when the absolute value of the average bias is used to calculate linking accuracy criteria instead of the RMSE

*C*_*R*_	*C*_*I*_= 1	*C*_*I*_= 2	*C*_*I*_= 3	*C*_*I*_= 4	*C*_*I*_= 5
Distribution 1					
1	.1023(.0999)	**.0774**(.0815)	**.0825**(.0829)	**.0722**(.0803)	**.0700**(.0745)
2	.0945(.0693)	**.0536**(.0685)	**.0522**(.0582)	**.0512**(.0615)	**.0440**(.0479)
3	.1044(.0879)	**.0628**(.0682)	**.0423**(.0545)	**.0430**(.0563)	**.0392**(.0517)
4	.0817(.0676)	**.0437**(.0560)	**.0359**(.0582)	**.0332**(.0375)	**.0282**(.0448)
5	**.0831**(.0888)	**.0392**(.0579)	**.0330**(.0477)	**.0380**(.0462)	**.0295**(.0486)
Distribution 2					
1	**.0603**(.0999)	**.0368**(.0815)	**.0343**(.0829)	**.0410**(.0803)	**.0387**(.0745)
2	**.0530**(.0693)	**.0357**(.0685)	**.0361**(.0582)	**.0269**(.0615)	**.0267**(.0479)
3	**.0421**(.0879)	**.0367**(.0682)	**.0256**(.0545)	**.0286**(.0563)	**.0265**(.0517)
4	**.0435**(.0676)	**.0315**(.0560)	**.0226**(.0582)	**.0206**(.0375)	**.0183**(.0448)
5	**.0528**(.0888)	**.0247**(.0579)	**.0223**(.0477)	**.0197**(.0462)	**.0149**(.0486)
Distribution 3					
1	.1108(.0999)	.0829(.0815)	**.0719**(.0829)	**.0732**(.0803)	**.0650**(.0745)
2	.0996(.0693)	.0702(.0685)	**.0566**(.0582)	**.0451**(.0615)	**.0438**(.0479)
3	.0931(.0879)	**.0564**(.0682)	**.0462**(.0545)	**.0442**(.0563)	**.0347**(.0517)
4	.0791(.0676)	**.0474**(.0560)	**.0469**(.0582)	**.0294**(.0375)	**.0335**(.0448)
5	**.0658**(.0888)	**.0452**(.0579)	**.0344**(.0477)	**.0347**(.0462)	**.0307**(.0486)
Distribution 4					
1	.1091(.0999)	.0835(.0815)	.0838(.0829)	**.0663**(.0803)	**.0631**(.0745)
2	.0879(.0693)	.0725(.0685)	**.0543**(.0582)	**.0470**(.0615)	**.0433**(.0479)
3	.0898(.0879)	**.0584**(.0682)	**.0403**(.0545)	**.0395**(.0563)	**.0341**(.0517)
4	.0836(.0676)	**.0487**(.0560)	**.0428**(.0582)	**.0260**(.0375)	**.0297**(.0448)
5	**.0860**(.0888)	**.0461**(.0579)	**.0300**(.0477)	**.0288**(.0462)	**.0292**(.0486)

## Conclusions

To examine one basis for the numbers of common raters and tasks required for high-accuracy test linking, we analyzed factors affecting test-linking accuracy for IRT-based performance tests using common raters and tasks. Specifically, we assumed that test-linking accuracy depends on three factors: 1) distributions of examinee abilities and characteristics of raters and tasks, 2) numbers of examinees, raters, and tasks, and 3) rates of missing data. We then performed simulation experiments to evaluate test-linking accuracy while varying these factors and numbers of common raters and tasks. From the results of these experiments, we discussed the numbers of common raters and tasks required for high-accuracy test linking for each condition set of each factor.

The experimental results for small- and mid-scale tests, in which the maximum number of examinees is 100, revealed the following:

1. In situations with no missing data, when the between-test ability distribution difference is relatively small, adequate test linking is possible with only one common rater and one common task. Even if the differences increase, two common raters and tasks are sufficient to ensure test-linking accuracy. We also showed that the extent of required commonality further increases when distributions of rater and task characteristics differ between tests, suggesting the importance of managing tests such that their characteristics are as equivalent as possible.
2. Increased numbers of examinees, raters, and tasks tend to decrease linking accuracy, but this effect is small under the small- or mid-scale settings. We found that we need only one common rater and one common task for small-scale settings, and two common raters and tasks are sufficient even for mid-scale settings.
3. As the rate of missing data increases, numbers of common raters and tasks must be increased. We showed that we need about *C*_*I*_ + *C*_*R*_ = 6 at minimum in cases of high rates of missing data.

An interesting observation from these results is that the required numbers of common raters and tasks are substantially smaller than those suggested by Linacre (2014). This is a nontrivial finding because it is practically important to minimize the numbers of common raters and tasks while maintaining desired test linking accuracy, as described in “Introduction”. Note that as discussed in “Effect of changes in characteristics of common raters and tasks”, in practice we may need to provide a safety margin by preparing slightly more common raters and tasks than as suggested above, to account for cases where rater and task characteristics change. The analysis in “Effect of changes in characteristics of common raters and tasks” also indicates the importance of carefully managing tests to ensure that changes in rater and task characteristics remain as small as possible, thereby lowering the required numbers of common raters and tasks.

This study further showed that under large-scale test settings, larger numbers of common raters and tasks than this standard by Linacre (2014) may be required, due to the large increase in numbers of examinees and raters and the larger rate of missing data.

The tendency for required commonality shown in this study is similar to that in several other studies of objective test linking (Kaskowitz and de Ayala, 2001; de Ayala, 2009; Ryan & Rockmann, 2009; Kolen & Brennan, 2014). Those studies suggest that the required number of common items is about 20–50% of the total test items for small- or mid-scale tests, and that even more are required for large-scale tests. Moreover, it is known that very few common items is adequate under some simulation settings (Kolen & Brennan, 2014). Our experimental results also show a similar tendency. Concretely, the results for the baseline setting (distribution 1) with missing data or with changes in characteristics of common raters and tasks, which will likely be an approximation of actual settings, suggest that we need about *C*_*R*_ + *C*_*I*_ = 5 or 6 at minimum, which corresponds to 25–30% of the total number of raters and tasks, *R* + *I* = 20. Also, the required commonality tends to increase as the test scale increases. Moreover, very few common raters and tasks (e.g., *C*_*R*_ = 1 and *C*_*I*_ = 1) are suggested to be adequate under some conditions.

As discussed above, required numbers for common raters and tasks depend strongly on settings. We therefore suggest that when designing performance tests, test administrators should verify linking accuracy following the experimental procedures presented in this study. See the *Open Practices Statement* regarding the programs we developed.

Note that this study does not focus on how to select common raters and tasks, despite this issue being important in practice. Several studies of objective test linking have suggested that common items are expected to be a subsample of the whole test (Kolen & Brennan, 2014; Ryan & Rockmann, 2009; Fink, Born, Spoden, & Frey, 2018; Born, Fink, Spoden, & Frey, 2019; Kim, Choi, Lee, & Um, 2008; Michaelides & Haertel, 2014). Specifically, it is commonly suggested that distributions of common-item parameters should be similar to the item parameter distribution in the whole test. In our study, common raters and tasks can be considered as samples from reference populations of raters and tasks because they are randomly drawn from a base test in which raters and tasks are sampled from the reference populations. Parameter distributions of common raters and tasks are thus theoretically consistent with those of raters and tasks in the whole test. Previous studies also showed that in practice we may require consideration of various factors, such as balance of item content and locations of the common items within a test. While these points will also be important for performance test linking, we will examine them in future works.

We will also examine other linking designs, such as those based on common examinees and those that simultaneously link more than two tests. Furthermore, although this study evaluated test-linking accuracy through simulation experiments, we hope to conduct experiments using actual data. Further investigations of linking accuracy under recent, more advanced MFRM extensions are also needed.

## Footnotes

1 Note that in this study, the term *task* represents a performance task, while *item* or *test item* represents various test-item types, including performance tasks and objective test questions.
